# OpenFungi: A Machine Learning Dataset for Fungal Image Recognition Tasks

**DOI:** 10.3390/life15071132

**Published:** 2025-07-18

**Authors:** Anca Cighir, Roland Bolboacă, Teri Lenard

**Affiliations:** 1 Department of Microbiology, George Emil Palade University of Medicine, Pharmacy, Science and Technology of Târgu Mureş, 38 Gheorghe Marinescu, 540139 Târgu Mureş, Romania; 2 Department of Electrical Engineering and Information Technology, George Emil Palade University of Medicine, Pharmacy, Science and Technology of Târgu Mureş, 1 Nicolae Iorga, 540142 Târgu Mureş, Romania; roland.bolboaca@umfst.ro (R.B.); teri.lenard@umfst.ro (T.L.)

**Keywords:** fungal identification, fungal image database, machine learning, convolutional neural network

## Abstract

A key aspect driving advancements in machine learning applications in medicine is the availability of publicly accessible datasets. Evidently, there are studies conducted in the past with promising results, but they are not reproducible due to the fact that the data used are closed or proprietary or the authors were not able to publish them. The current study aims to narrow this gap for researchers who focus on image recognition tasks in microbiology, specifically in fungal identification and classification. An open database named OpenFungi is made available in this work; it contains high-quality images of macroscopic and microscopic fungal genera. The fungal cultures were grown from food products such as green leaf spices and cereals. The quality of the dataset is demonstrated by solving a classification problem with a simple convolutional neural network. A thorough experimental analysis was conducted, where six performance metrics were measured in three distinct validation scenarios. The results obtained demonstrate that in the fungal species classification task, the model achieved an overall accuracy of 99.79%, a true-positive rate of 99.55%, a true-negative rate of 99.96%, and an F1 score of 99.63% on the macroscopic dataset. On the microscopic dataset, the model reached a 97.82% accuracy, a 94.89% true-positive rate, a 99.19% true-negative rate, and a 95.20% F1 score. The results also reveal that the model maintains promising performance even when trained on smaller datasets, highlighting its robustness and generalization capabilities.

## 1. Introduction

Fungi are eukaryotic microorganisms that can be found everywhere in the environment. They can be saprophytic or plant, animal, and human pathogens, with significant impact on the medical and agricultural fields. The fungal kingdom is very wide, encompassing between 1.5 and 5 million species, but very few of them are primary pathogens able to cause infection in both immunocompetent and immunosuppressed individuals. The majority of filamentous fungi are opportunistic pathogens, requiring specific host conditions to cause infections (i.e., immunosuppression). Therefore, the most affected population is the one with different risk factors, such as solid organ transplant, hematological diseases, stem cell transplantation, HIV, or diabetes mellitus [1].

As life expectancy and, subsequently, the number of risk factors increase, so does the number of infections by filamentous fungi [2]. Mold infections are challenging to treat; therefore, fast and accurate diagnosis is mandatory. Classical laboratory diagnostic methods include culture, microscopy, and histopathological examinations, while more modern methods rely on serology and molecular testing. As newer methods are not yet available widely, culture and microscopy still remain the gold standard for diagnosis, but they come with challenges, as culturing filamentous fungi is time-consuming and requires trained professionals for accurate identification [3].

This can be improved in the future by multidisciplinary approaches. After the technological booms of the past decade, a particular subject that everyone started to talk about is the automation benefits that Artificial Intelligence (AI) and machine learning (ML) bring. convolutional neural networks (CNNs), a class of deep learning algorithms, have proved to have remarkable success in different tasks involving image classification, especially in the medical [4,5], agricultural [6,7], and food [8] fields. In fungal identification, CNNs have significantly improved classification accuracy, especially in scenarios with large, labeled datasets [9].

Currently, there are several limitations that must be addressed regarding ML applications in filamentous fungal identification. Firstly, the models used for image classification are too large (e.g., deep neural networks), requiring high-quantity and -quality datasets, computational resources, and extended time for data processing and analysis. Furthermore, progress in improving these models is sparse and slow, as a lot of authors currently publishing new studies do not make the data used available.

To address these issues, the current paper comes forward to narrow the gap in the multidisciplinary domain of ML and microbiology with four distinct contributions:

1. An open database named OpenFungi is made available: it contains 1249 high-quality macroscopic and microscopic images of different fungal genera. The OpenFungi dataset contains five genera of filamentous fungi: *Aspergillus* spp. (further subdivided into *Aspergillus* section *Flavi* and *Aspergillus* section *Nigri*), *Penicillium* spp., *Rhizopus* spp., *Alternaria* spp., and *Fusarium* spp. Furthermore, mixed cultures of different filamentous fungi are included in the database. To avoid the lengthy time necessary to isolate fungal strains from patients and obtain ethical approvals, all the presented fungal genera were isolated from food products, specifically green leaf spices and different cereals commercialized in Romanian supermarkets.
2. The quality of the dataset is demonstrated through the use of a CNN for an image classification problem.
3. This paper demonstrates and advocates that through a curated dataset, small CNNs can yield promising results with high accuracy and close-to-zero false positives even in balanced datasets.
4. Along with the open dataset, the simple code used to produce these results is made openly available to the research community.

Taking into consideration that there are a very high number of filamentous fungal genera, and even more species, building a dataset that encompasses all of them is a challenging task. Therefore, the current article aims to be a starting point in automating the identification of common fungal pathogens that can be found in food products, as well as in clinical practice. This means that the current database is in its first version and will further improve over time, adding new genera and species as the work goes further.

The rest of the paper is structured as follows: Section 2 is split in two main parts. First, the data generation process is presented, covering fungal cultivation and image capturing. Second, the CNN model formal fundamentals are presented. Afterwards, in Section 3, the experimental results and model performance are presented. Section 4 covers a series of discussions, with the paper concluding in Section 5.

## 2. Materials and Methods

### 2.1. Filamentous Fungal Strains—Cultivation and Identification

The first major step in creating the OpenFungi dataset included filamentous fungal strain cultivation and identification. Fungal cultures were obtained by processing two different types of food products, namely, green leaf spices and cereals. Green leaf spices were cultivated and analyzed for their fungal load and mycotoxin content in a previously published study [10], while the ones originating from cereals are part of the current and ongoing work.

As described in a prior work [10], in the case of green leaf spices, 21 packages weighing 7–10 g of product were bought from a common supermarket. The packages consisted of seven different green leaf spices from three different producers, from three different price ranges: oregano (*Origanum vulgare*), thyme (*Thymus vulgaris*), basil (*Ocimum basilicum*), lovage (*Levisticum officinale*), parsley (*Petroselinum crispum*), dill (*Anethum graveolens*), and rosemary (*Rosmarinus officinalis*). For cereals, 12 packages weighing 200–500 g were purchased from the same supermarket, classified as natural, with no preservatives, or bio and cereals that are more processed and considered unhealthy. Several types of cereals were analyzed, such as oats (e.g., rolled and steel-cut), oat bran, barley, rye, corn flakes (e.g., with and without chocolate and sugar), wheat, mixed cereals, muesli, chocolate, and cinnamon flakes.

Different fungal strains were isolated and identified using a Standard plate count method [10]. For each food product, 5–10 g (i.e., 5 grams for spices and 10 g for cereals) was weighed in a sterile container by using an analytical balance (Kern, Balingen, Germany) and then mixed with 90 mL of sterile saline. The suspension was then blended using a magnetic stirrer for 10 min at 360 rotations/min. Subsequently, the mixture was left on the table for sedimentation at room temperature for approximately 30 min.

From the supernatant, a 1/100 dilution solution was mixed first, to facilitate colony counting, fungal isolation, and identification in the case where the analyzed samples contained a very high fungal load. To make a 1/100 dilution, in a separate 1.5 mL sterile Eppendorf tube, 990 μL of sterile saline was mixed with 10 μL of the supernatant. Afterwards, the samples and dilutions were seeded on culture media. The media were chosen according to their specificity for filamentous fungi and incubated at set temperatures suitable for molds (Table 1).

Each culture medium was numbered accordingly per sample. From each supernatant and dilution, 100 μL of solution was added to every medium by using an automatic pipette and sterile tips. The inoculum was then spread using a sterile 10 μL loop in three directions. Subsequently, the medium was incubated under the previously established conditions: 25 °C for 24–72 h, extended to 7 days, when necessary. If mixed cultures were present, the different strains were isolated on PDA and incubated again at 25 °C for 24–72 h to obtain a pure culture. Identification to the level of genus was performed from the cultures obtained, based on the macroscopic aspect on PDA correlated with microscopic examinations. The studied macroscopic traits included colony invasivity, growth rate, culture granularity, and color.

For microscopic examinations, the scotch tape method with lactophenol cotton blue staining (Sigma-Aldrich, St. Louis, MO, USA) was used. In this special staining used for molds, lactophenol inactivated the spores, while the cotton blue colored all the structures in blue, except for older cultures and dematiaceous molds, which have their own characteristic brown pigment. For each of the studied strains, specific structures were searched for in microscopy: septate hyphae and aspergillar heads in *Aspergillus* spp. and non-septate hyphae for inferior molds, such as *Rhizopus* spp. The characteristics of each filamentous fungi were compared with studies from the literature for identification to the level of genus.

### 2.2. Image Capturing

To create the dataset, pictures of both the mixed and pure cultures were taken at set intervals of time, 24, 48, and 72 h, prolonged if necessary (i.e., for slower-growing species) for up to seven days. For the pure cultures, only images on PDA were used. For the mixed cultures, pictures from all three culture media were used: PDA, Czapek, and RBCA. To facilitate identification, pictures of microscopic examinations in lactophenol cotton blue staining were also included (10× and 40× magnification). The images included in the database were collected over a period of five years (2021–2025).

### 2.3. Image Classification

CNNs are a class of deep learning algorithms that have shown significant performance in automated image recognition in numerous fields, including medical imaging. Unlike traditional artificial neural networks, CNNs are made up of sequential processing layers designed to automatically extract and learn relevant features from input images. Typically, CNNs include three types of layers: convolutional layers, pooling layers, and fully connected layers. Convolutional layers apply small filters (also known as kernels) over the input image channels (e.g., RGB) to detect local patterns such as edges, lines, and textures. These filters are slid across the image, performing dot products at each location to capture meaningful visual features. Pooling layers are applied after convolutional layers and have two main purposes. First, they reduce the spatial resolution of feature maps, which reduces the computational load. Second, they help the model retain the most important image features, improving the ability to recognize patterns regardless of their location. Finally, fully connected layers integrate the features learned throughout the image. Each neuron in these layers is connected to every neuron in the previous and subsequent layers, allowing the network to make global decisions based on local characteristics [11]. The output layer consists of *k* neurons, where *k* corresponds to the number of categories; in this study *k* denotes the number of images of fungal species.

As with other supervised learning approaches, CNNs are trained using labeled data, where each image in the training set is associated with a known category. After training, the model can classify previously unseen images, since these images share the same features as the categories seen during training, with a certain degree of confidence. In practical terms, a trained CNN is capable of recognizing and categorizing a previously unseen image based on the similarity to the patterns it has learned to associate with each fungal class. CNNs, like standard artificial neural networks, are trained using a technique called backpropagation. During this process, the model’s internal parameters (also known as weights) are updated iteratively to minimize a loss function, which quantifies how far the model’s predictions are from the true labels. Training occurs over multiple iterations, known as epochs, and the objective is to reduce the number of misclassified images while maintaining the ability to generalize to new, unseen clinical cases.

To evaluate the performance of the CNN in classifying and identifying fungal species from images, the following experiments were performed:

1. The CNN model was trained using 80% of the images that represent all fungal species included in the study and evaluated on the remaining 20%. This split is commonly used in machine learning research to measure performance in previously unseen cases.
2. To account for the variability introduced by the random initialization of the model parameters and the random shuffling of the dataset, training and evaluation were repeated across multiple independent trials. For each trial, a new random seed was applied, and the model was re-trained. The mean and standard deviation of the performance metrics were then computed in all repetitions of the experiment to evaluate the robustness of the model.
3. To identify the relationship between the size of the training dataset and the classification performance, the model was trained on progressively larger sets of data. Starting with 10% of the available images, the training set was incrementally increased by 10% in each trial, while the remaining images were used for testing. This experiment aimed to determine the minimum dataset required for the model to achieve acceptable performance.
4. To address class imbalance in the dataset, several techniques were employed and validated, including oversampling and subsampling. A baseline for the number of images per class was set as the average between the maximum and minimum overall available samples. For the majority classes, the number of samples was reduced using subsampling (e.g., randomly selecting only a portion of the images); meanwhile, for the minority classes, the number of samples was increased using oversampling with augmentation techniques (e.g., random rotations, zoom, contrast changes, and horizontal flips). After applying these techniques, the resulting classes were perfectly balanced.

To evaluate the performance of the CNN model in classifying fungal species from images, the following relevant metrics are used:

- Sensitivity, or True-Positive Rate (TPR)—The proportion of true-positive cases correctly identified by the model. This shows the model’s ability to detect a fungal species when it is present in the image. Depending on the applicability field, sensitivity is also known as TPR or Recall.
- Specificity, or True-Negative Rate (TNR)—The proportion of true negative cases correctly identified. This indicates how effective the model is in avoiding false positive results.
- Positive Predictive Value (PPV)—The proportion of positive classifications that are truly positive. This metric corresponds to precision.
- Negative Predictive Value (NPV)—The proportion of negative classifications that are truly negative, showing the model’s ability to correctly exclude a specific fungal presence.
- F1 score (F1)—The harmonic mean of sensitivity and PPV, which provides a balanced measure in the presence of class imbalance.
- Accuracy (ACC)—The overall proportion of correctly classified cases in all categories.

Except for accuracy, all other performance metrics are computed both overall and separately for each fungal genus (e.g., each class). The equations for each metric are provided in the Results Section (Section 3).

### 2.4. Dataset Description

As described above, two datasets were created for this study: one with macroscopic images and one with microscopic images of fungal species. The macroscopic dataset contains a total of 678 high-quality images (4624 × 3472 pixels), including a mixed category with various combinations of genera. This dataset includes pictures of the following genera: *Alternaria* spp., *Aspergillus* spp. (further divided into *Aspergillus* section *Flavi* and *Aspergillus* section *Nigri*), *Fusarium* spp., *Rhizopus* spp., *Penicillium* spp., and mixed cultures. The microscopic dataset consists of 571 high-quality images (2560 × 1920 pixels) and covers five fungal genera as follows: *Alternaria* spp., *Aspergillus* spp. (divided into *Aspergillus* section *Flavi* and *Aspergillus* section *Nigri*), *Fusarium* spp., *Rhizopus* spp., and *Penicillium* spp.

**Figure 1:**
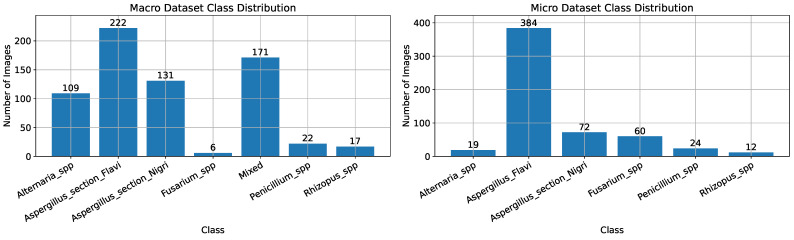
Class distribution for macroscopic (Macro) dataset (**left**) and microscopic (Micro) dataset (**right**).

**Figure 2:**
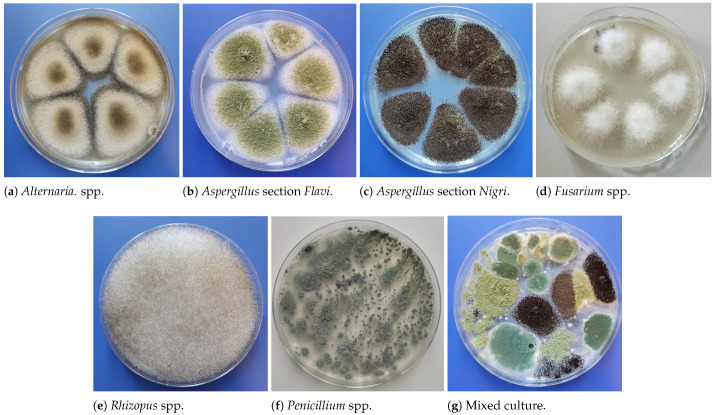
Example images of macroscopic culture aspects from the dataset.

**Figure 3:**
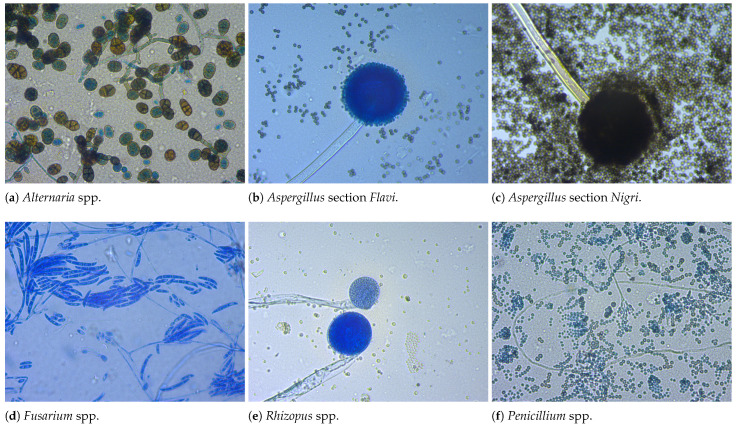
Example images of microscopic aspects from the dataset.

The distribution of cases per fungal genera, for both macroscopic and microscopic image datasets, is shown in Figure 1. As seen, the datasets are imbalanced, which means that the number of entries differs among the fungal genera. Examples of the images contained in both datasets are shown in Figure 2 and Figure 3. The OpenFungi dataset, with both the microscopic and macroscopic images, can be accessed online (https://doi.org/10.5281/zenodo.15692069, accessed on 10 July 2025), and it is distributed under the Creative Commons Attribution 4.0 International license.

## 3. Results

### 3.1. Experimental Setup

The experimental setup included a desktop computer running a Linux operating system with kernel version 5.15.0. The setup included an AMD Ryzen 5 7600X CPU running at 4.7 GHz, 32 GB of DDR5 RAM, and an RTX 4070 SUPER 12 GB GDDR6X GPU. In terms of implementation, Python 3.10.12 was used with the following modules: numpy 2.1.3, tensorflow 2.19.0, and keras 3.9.2. The source code used to produce the results is distributed as jupyter notebooks and is made available under the MIT License on GitHub (https://github.com/rolandbolboaca/CNN_Fungi_classification, accessed on 10 July 2025).

The best hyperparameters for the CNN model were obtained using the grid search optimization method [12]. This approach involves testing the CNN model with Cartesian products of hyperparameters values from fixed given ranges. The following hyperparameter ranges were explored during model optimizations: CNN hidden layers [1, 5], including convolutional layers [1, 3] and fully connected layers [1, 2]; number of hidden units for each layer [8, 128]; input layer size [(64, 64), (328, 328)]; learning rate [0.001, 0.1]; and number of training epochs [10, 100]. The best results, which also include the model’s architecture, are provided in the following for accurate reproducibility of our experiments: We used an input layer of size (128, 128), where each image was resized to 128 × 128 pixels; two convolutional 2D layers that encompassed 32 and 64 kernels, respectively, each with a 2D max pooling layer; and a single fully connected dense layer composed of 64 standard neurons. All layers utilized the Rectified Linear Unit (ReLU) activation function. A final fully connected output layer that utilized softmax activation for classification was considered. The model was trained using the Adam [13] optimizer using a sparse categorical cross-entropy loss function with the accuracy metric. The model was trained for 35 epochs with a batch size of 32 and a learning rate of 0.001. The default training size was set to 80%, which involves selecting 80% of the entire dataset for training (and validation during training) and allowing 20% to remain for testing. For validation during training, 1% of the entire dataset was used.

During preprocessing, the dataset was normalized in the [0, 1] range. The images were not cropped, as the CNN model is capable of extracting relevant features without the need for manual adjustments. The same approach was applied to variations in lighting, focus, and background. As mentioned above, for additional experimentation with class imbalance, the images were augmented using random rotations, zoom, contrast adjustments, and horizontal flips.

To avoid data leakage, that is, using data that were seen during training for model validation, the following steps were taken: First, the entire dataset was loaded into memory. Second, all the images were shuffled. Third, the training data (e.g., data used only for model training), the test data (e.g., data used only for test purposes), and the validation data (e.g., used during model training to avoid overfitting) were clearly separated by extracting the above-mentioned percentages, in order training, testing, and finally validation, without any set overlap. After creating the subsets, the splits were locked, and no other data mixing or modifications were made.

### 3.2. Model Performance

The performance of the CNN models was measured using the metrics described in the previous section, namely, TPR, TNR, PPV, NPV, F1, and ACC, as they appear in Table 2. As stated above, except for ACC, all other performance metrics were computed both overall and separately for each fungal genus.

**Figure 4:**
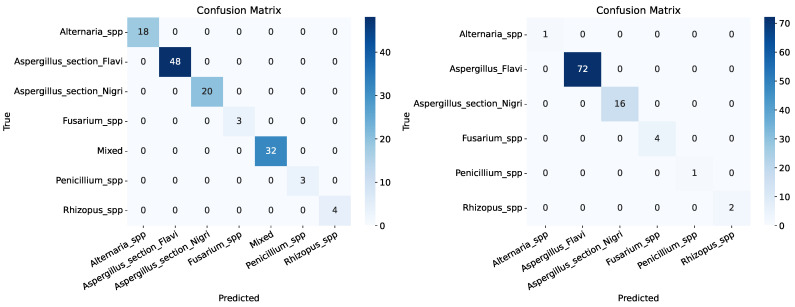
Confusion matrices illustrating the classification performance of the CNN model on the macroscopic test dataset (**left**) and on the microscopic test dataset (**right**).

On both the macroscopic and microscopic datasets, the results of the first experiment, in which the model was trained on 80% of the images and tested against the remaining 20%, were 100% for all metrics both overall and per fungal species. These results are indicators that the model correctly classified all test cases without any errors. Figure 4 illustrates the confusion matrix for this experiment. As observed, the model correctly assigned the class to each of the pictures, even with a few training cases, such as *Fusarium* spp., *Rhizopus* spp., and *Penicillium* spp. Although these results indicate that the CNN model obtained 100% accuracy, which is usually obtained only under specific controlled conditions, this experiment did not consider sensitivity to random initializations and image shuffling.

In the second experiment, the model was reinitialized and re-trained, and the dataset was shuffled before creating the training and test subsets. This process was repeated 100 times. The results of this experiment, including overall and per-fungal species results, for both datasets are shown in Table 3 and Table 4. As it can be observed, there are performance variations in all metrics. This behavior is expected, as any neural network-based algorithm is sensitive to initial conditions, that is, to the randomizations of the initial parameter values. In addition, the effects of data shuffling are also reflected in the results. Although the previous experiment revealed notable scores of 100%, some limitations of this model are shown here. Nonetheless, on both datasets, even with performance variations, the CNN model obtains notable performance in all metrics.

As shown in Table 3, the overall results on the macroscopic dataset were consistently high, with values above 99.55% across all metrics and standard deviations remaining below 1.8%. The best per-class performance was achieved by *Fusarium* spp., with 100% scores on all metrics. The lowest performance was recorded for *Rhizopus* spp., with a TPR of 98% and slightly lower but still high values for the remaining metrics. Despite minor variations across classes, all other minimum values exceeded 99%, and standard deviations were consistently low (under 9%), indicating high stability.

Compared with the previous scenario, performance on the microscopic dataset, shown in Table 4, was generally lower for all metrics. These results can be attributed to the increased variability and heterogeneity of the micro-level data, which include more subtle inter-class differences and possible class overlap. The most noticeable differences were observed in the TPR, F1 score, PPV, and ACC values. In general, the CNN model achieved an ACC of 97.82% (±5.7%), a TPR of 94.89% (±14.12%), and a PPV of 96.44% (±12.03%). Per class, the highest scores were obtained for *Aspergillus* section *Flavi*, with a TPR of 98.48% and an F1 score of 99.95%, with low standard deviations. The lowest per-class results were observed for *Penicillium* spp., with a TPR of 89.60% (±23.23%) and an F1 score of 91.92% (±20.39%). These lower performances are attributed to low sample sizes for *Alternaria* spp., *Penicillium* spp., and *Rhizopus* spp. Nonetheless, as shown in Table 4, the CNN model obtained reliable and robust results with values above 94.89% across all metrics for the per-class averaged (macro-average) scenario.

Overall, the models achieved strong performance across both macroscopic and microscopic datasets, with high average F1 scores and low variance for the majority of fungal genera. In particular, *Aspergillus* spp. showed consistent results across multiple metrics, likely due to their relatively distinct visual features and better representation in the dataset. In contrast, lower scores and higher variability were observed for *Penicillium* spp. and *Rhizopus* spp., especially in the microscopic dataset. This drop can be attributed to the visual overlap between genera and possibly class imbalance, as these categories had fewer training instances, making them more prone to misclassification.

**Figure 5:**
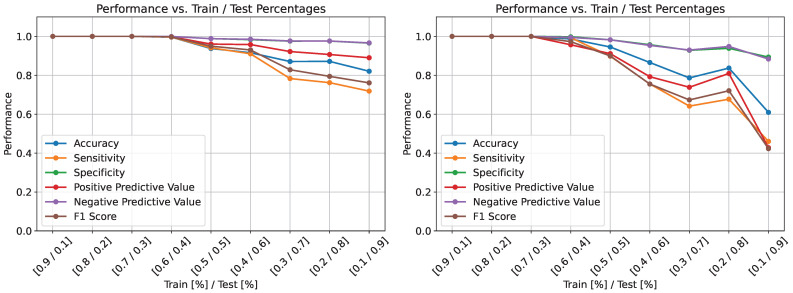
CNN performance values with regards to training/test percentages on the macroscopic dataset (**left**) and microscopic dataset (**right**).

The previous results highlight the fungal classification performance of the CNN model on both the macroscopic and microscopic datasets. However, in those experiments, the CNN model was trained on 80% of the data and tested against the remaining 20%. In many clinical and laboratory settings, obtaining new high-quality data can be difficult, time-consuming, or even infeasible. As mentioned above, in the third experiment, the model was trained on decremental percentages of the data in the [90%, 10%] range. The results of these experiments are illustrated in Figure 5.

On the macroscopic dataset, the model’s performance is maintained at almost 100% even when trained on 60% of the data. After the 60% mark, the performance of the model slowly decreases, reaching 95% accuracy, 92% sensitivity, 95% F1 score, and 99% PPV when trained on 50% of the data. The lowest performance is obtained when the model is trained on 10% of the cases, with 82% accuracy, 95% specificity, 95% NPV, 72% sensitivity, and 76% F1 score. As observed in Figure 5, when the number of fungal images used for the training of the CNN model varies between 60% and 10%, overall performance decreases.

On the microscopic dataset, the model performance remains 100% in all metrics when trained on 90%–70% of the data. Similarly to the previous case, the model performance slowly decreases with the training size. The lowest accuracy of 60% is obtained when the model is trained on only 10% of the data. In terms of sensitivity and F1 score, the lowest values obtained are between 40% and 50% when the CNN model is trained on only 10% of the fungal images. An interesting observation can be made after the 40% training size mark, when there is a slight increase in performance in all metrics. These results show that the performance of the CNN model is highly dependent on the diversity and the number of cases it sees during training. Although this is an important observation, it is also well known that machine learning algorithms depend heavily on data quality and variety and random initializations (as shown in the results of the previous experiment).

For the class imbalance experiment, the application of data undersampling and oversampling techniques resulted in perfectly balanced classes, with a total of 798 samples for the macroscopic scenario and 1188 samples for the microscopic scenario. The results of this experiment are shown in Table 5 and Table 6, corresponding to the macroscopic and microscopic datasets, respectively. The numerical values in these tables represent the averages of 100 repetitions with random model initializations.

On the macroscopic dataset, there was a slight decrease in overall and per-class performance when augmenting the dataset, with a 1% decrease in the TPR, a 0.2% decrease in the TNR, and a 1% decrease in terms of the F1 score, as observed when analyzing Table 3 and Table 5. In contrast, on the microscopic dataset, a slight increase in performance was observed overall and per class, with a 2% increase in the overall TPR and F1 score. Apart from the *Aspergillus* section *Flavi* class, after data augmentation, the performance slightly increased for each of the remaining classes. The overall accuracy remained almost similar in both approaches, as observed in Table 4 and Table 6.

Augmentation particularly benefited minority classes. For example, the F1 score for *Penicillium* spp. on the microscopic dataset increased from 0.9192 to 0.9615 and, for *Rhizopus* spp., from 0.9300 to 0.9782. Furthermore, the per-class TPR and PPV became more balanced across all categories, reducing the discrepancy between minority and majority classes. This suggests that the model learned more generalizable features for each genus after augmentation. The results also support the earlier observation that even with fewer training samples (e.g., by subsampling the majority classes), the model maintained notable performance.

The training and test times, measured in the setup mentioned above, were on average 130 s for training and 6 s for testing. In general, for neural network-based algorithms, the running times are considerably higher during training due to the iterative optimization steps performed (e.g., backpropagation), as detailed in the previous section. Training and test times are also dependent on the architecture on which the model is executed.

## 4. Discussion

Filamentous fungi are found everywhere in the environment, in soil, water, air, food, and all kinds of substrates. As they have high medical and economic impact [14], fungal identification is mandatory in a variety of fields, from healthcare to agriculture.

In recent years, the number of mold infections increased progressively due to several factors: the continuously growing number of people with immunosuppression due to different causes (solid organ transplantation, hematopoietic stem cell transplants, autoimmune diseases, etc.), prolonged life expectancy associated with multiple co-morbidities, different oncological diseases (especially hematologic malignancy), and more [15]. This further contributes to the idea that diagnosing such infections should be fast and accessible, as accurate and time-efficient treatment improves survival rates and antifungals work best when the fungal load and the risk of dissemination are still low [16].

Both in the clinical setting and in the laboratory, the diagnosis of fungal infections is very challenging, especially for filamentous fungi. Nowadays, as progress is being made at a rapid pace, sequencing is regarded as the gold standard for the diagnosis of fungal infections. But it has its limitations, due to complex sample preprocessing, high costs of testing, and lack of test standardization and is currently being saved for special cases, such as rarely isolated species [17]. A more accessible way that is still used worldwide is the classical method of diagnosis, which includes cultivating the mold on specific culture media and performing identification based on the macroscopic and microscopic aspects of the culture, which requires trained professional specialists in the field of mycology [18]. Especially in small laboratories, trained professionals are not available, delaying diagnosis. Here is where ML and AI come in.

In the field of medical mycology, AI and ML are set to change the way diagnosis is viewed. In fungal infections, diagnosis is slow, as filamentous fungi take a long time to grow on culture media, and sometimes challenging, due to the variability in macroscopic and microscopic morphology and the wide variety of genera and species that are known so far [19]. There is a variety of AI- and ML-based models that have been developed for all kinds of procedures from the diagnostic scheme:

- Analyzing datasets containing microscopic images of specimens for either Gram staining or potassium hydroxide (KOH) examinations [20,21];
- Fungal culture identification [9];
- Histopathological slides to differentiate between colonization and infection [22];
- The classification of fungal strains into different genera and phyla based on sequencing [23].

All tests are then validated through other methods (i.e., having a trained specialist confirm the results or using confirmatory tests such as polymerase chain reaction—PCR) [24]. In order for ML models to be trained and be able to help identify fungal isolates, high-quality data are mandatory. In a prior work [2], we analyzed a total number of 23,777 samples of respiratory samples and purulent secretions over a period of 10 years, with 68 of them being positive for mold infections. Taking this into consideration, collecting data for model training would have been really challenging and time-consuming. In order to address this situation, different filamentous fungal strains were isolated from food products. Furthermore, since the fungal strains were not isolated from patients, no ethical approval was necessary.

Filamentous fungi from food are a rare but known cause of invasive infections in immunocompromised patients. A comprehensive literature review by Benedict et al. from 2017 [25] found eight articles that describe filamentous fungal infections acquired from food products. Different fungal pathogens were involved in those infections, such as *Aspergillus fumigatus*, *Fusarium moniliforme*, and inferior fungi such as *Rhizopus* spp., *Mucor* spp., and *Absidia* spp. The suspected sources of those infections varied from fermented beverages to cooked rice, cereals, and dairy products. An interesting article published by Lazar et al. (2014) [26] presents the case of a man with poorly controlled diabetes and acute myeloid leukemia who contracted *Mucor circinelloides*, probably from a contaminated yogurt.

This provided us with an opportunity to create the database faster and with a wider variety of input data. Therefore, the model presented in this article, as well as the database that we provide, can be used by a wide range of people, from healthcare professionals trying to diagnose filamentous fungal infections to plant experts trying to identify pathogens destroying crops. The ML model at hand provides an easy and time-efficient alternative, compared with other deep learning models that are currently available in the literature. Furthermore, both the model used and the database are openly available and constantly updated to encourage further research in the field.

Several articles are available on fungal identification, but some of them have different advantages or limitations compared with the work at hand. An article published by Rahman et al. [27] in 2023 uses multiple CNN models, such as DenseNet, Inception ResNet, InceptionV3, Xception, ResNet50, VGG16, and VGG19, for the identification of both yeasts and molds from microscopic images. The study uses a database of 1079 images, including 89 genera, compared with our database, which includes 1249 images but only 5 genera. A limitation of this study is that the database has not been made available, discouraging future research on the topic. Furthermore, the authors mention that their models had challenges in differentiating between *Aspergillus* section *Nigri* and *Aspergillus* section *Flavi*, a problem that is addressed by the current model by using both macroscopic images from cultures and microscopic images. Regarding model performance, the model used by Rahman et al. with the best performance was the DenseNet CNN model, which had an accuracy of 75.91%, while the CNN model used in this work can reach an accuracy of almost 100%, with accuracy in the 70–80% range only when the CNN model was trained on a very small part of the available data (10%).

Another article, published by Mansourvar et al. [9] in 2025, analyzed three advanced deep learning architectures—ResNet50, DenseNet-121, and Vision Transformer (ViT)—and assessed their performance in identifying filamentous fungi solely based on macroscopic aspects of the culture. The most remarkable thing about the article is that the authors used a very large database for training and testing the models, as they took time-lapse pictures of 110 fungal species every 30 min for up to seven days. This resulted in very good input data for their models, as they finally used 26,451 images (10,027 for training, 4827 for testing, and 11,597 for validation). Compared with Mansouvar et al., the current article uses a smaller database but includes both images of fungal cultures in different stages of evolution and microscopic images, making it a very accessible tool. Another thing worth mentioning is that the authors made the dataset available upon request. Regarding accuracy, the models used by Mansourvar et al. also showed lower accuracy compared with the current model, with ViT as the best performing model, with an accuracy of 92.64%, followed by DenseNet-121 and ResNet50 with 86.77% and 76.75% accuracy, respectively.

Other studies in the area include an article published by Tsang et al. [28] in 2024, where they used deep learning models such as ResNet-18, Inception-v3, and DenseNet-121 to analyze 11,762 images (2813 training images, 2814 validation images, and 1240 test images) of four different species of *Aspergillus* (*Aspergillus flavus*, *Aspergillus fumigatus*, *Aspergillus niger*, and *Aspergillus terreus*), totaling 107 different fungal strains. While their database is larger than the one used in our study, it is not made available to other researchers. Furthermore, the study is limited to culture images of a very limited number of species of fungi, compared with the study at hand, which describes a wider variety of commonly found fungal genera. Regarding the deep learning models used, while they did considerably reduce the time to identification as opposed to classical diagnosis, they were still quite time-consuming when trained on large datasets (19 min), while the CNN proposed by us had a running time of 130 s for training. Furthermore, Tsang et al. only included in the database pictures of the four species seven days after cultivation, thus ensuring proper sporulation and easier recognition by the deep learning models. In our case, there are different pictures available starting from 24 h up to seven days after culture inoculation, facilitating earlier recognition of the genera by the ML model while still maintaining the really good training time mentioned before. Additionally, our model had a similar but slightly better accuracy (99%) compared with the values in Tsang et al.’s article (97.96%, 97.01%, and 96.88% for DenseNet-201, Inception-v3, and ResNet-18, respectively).

Javidan et al. [29] proposed a method for the automated identification of fungal species originating from tomatoes by analyzing microscopic images of spores. Starting from an image dataset, the authors employed a feature extraction methodology based on the Butterfly Optimization Algorithm. The most relevant features obtained represent the color, shape, and texture. Subsequently, a Random Forest classifier was trained on the resulting features. The results obtained show an accuracy of 98% on the full feature set and one of 95% on the top eight selected features. From the point of view of experimental assessment, the authors compared their approach with a standard CNN and an EfficientNet model, which yielded less accurate results.

Lastly, Hassan et al. (2025) [30] leveraged a YOLOv8 model together with a custombuilt dataset to identify five *Aspergillus* species, namely, *A. terreus*, *A. fumigatus*, *A. flavus*, *A. welwitschiae*, and *A. austwicki*. The authors used an object detection model trained on 337 images, with different variations in image background, light, angle, and spore density. Subsequently to fungal isolation and molecular verification, each image was annotated and processed through the YOLOv8 model. The results obtained show a mean average precision of 90% and a sub-2 second inference time. A limitation present in the study is reflected in the lack of variety of fungal species and its focus only on microscopic images.

Despite the novelties it brings, this article has several limitations. From a microbiological standpoint, the main problem is that fungi are only identified to the level of genus, and not species, based on classical laboratory methods of diagnosis. Although this could be improved, from a clinical point of view, the most important thing when administering treatment is knowing the genus as quickly as possible to be able to predict any natural resistance to the currently used antifungals. As some species are more common than others, both in natural environments and in patient samples, the presented datasets are inherently unbalanced. In other words, there is a natural discrepancy in the number of available per-species samples in both the microscopic and macroscopic scenarios. Nonetheless, this issue was addressed in this paper through the use of data balancing techniques, which produced promising results and further demonstrated the robustness of both the dataset and the CNN model used.

To summarize the current discussion, Table 7 presents a feature-based comparison with the most relevant related studies. There are several points that differentiate the current work from the studies in Table 7. From the point of view of the model used, we observed that most studies are inclined to used pre-trained models, compared with our smaller model, which is a standard CNN, purposely trained for macroscopic and microscopic mold identification. In terms of scope, our work differentiates itself as it addresses both macroscopic and microscopic identification. From the studies analyzed, the data on which experiments were conducted in related studies are closed and only available upon request. Source code availability is mixed, with works such as [28] providing a reference to the project’s code. Lastly, the metrics used in related studies mostly follow best practices in terms of model evaluation.

## 5. Conclusions

Research data on filamentous fungi identification are still sparse. While newer methods like sequencing and MALDI-TOF MS are slowly replacing classical methods of diagnosis such as culture and microscopy, machine learning and AI could play a significant role in reducing the time to diagnosis even more. More and more data on different models have been published in the recent literature, but there are still two things missing: a simple and time-efficient algorithm that can process images rapidly and with high accuracy and an open database with a wide variety of microscopic and macroscopic images that can be accessed by researchers and improved in the future. This is why the article at hand aims to fill this gap in the literature by proposing an easy-to-use and rapid CNN model that accurately identifies five different filamentous fungal genera, as well as an open database that contains a variety of molds to encourage further research and improvement.

### Future Work

In terms of future work, there are two main directions available at this moment: (i) extending the database with more relevant data and fungal species at macroscopic and microscopic levels and (ii) evaluating, testing, and improving ML models for advanced fungal identification. For point (i), a similar approach to data collection to the one of Mansourvar et al. [9] is envisioned. By establishing a consistent data collection protocol, the aim is to create a dataset that contains images of the growth process of fungal genera. Compared with static images, as it is the case in this work, such a dataset introduces the temporal aspect in the problem. Since fungi take a long time to grow and develop their characteristic traits for identification, this means that the growth behavior can be constantly monitored and analyzed with the aim to extract and identify particular insights specific to each genus. Furthermore, as more time will be available to further develop this project, we will be able to introduce a variety of new fungal genera and species and progressively expand the dataset. The database updating process will be performed through Zenodo’s versioning system. The benefit of using Zenodo is that each version remains available, with its specific tag associated. This means that no data will be lost upon update. The second point (ii) is tightly linked with the first one. By having a look at the related studies [4,5], we observed that pre-trained models are frequently used in problems similar to the one solved in this work. While we advocate that keeping the model small and specific to a single purpose is better, future work plans involve pre-trained model testing and evaluation in combination with transfer learning [31]. Moreover, with the extension of the dataset, research indicates that models such as a long short-term memory in combination with CNNs could yield promising results. Nevertheless, decision systems with ensemble methods will be investigated to determine the possibility of combining the output of multiple small models (e.g., micro- and macro-models) into a single result.

## Figures and Tables

**Table 1:** Composition, incubation conditions, and purpose of the selected culture media used for isolation of filamentous fungi.

Culture Medium	Composition	pH	Incubation Conditions	Purpose
Potato Dextrose agar (PDA)—Oxoid, UK	Commercial PDA powder (20 g of dextrose, 15 g of agar, and 4 g of potato starch), distilled water, and Chloramphenicol	5.6 ± 0.2	25 °C for 24–72 h	Cultivation of both superior and inferior molds from food products
Czapek agar—Oxoid, UK	Commercial Czapek agar powder (agar, sucrose, sodium nitrate, dipotassium phosphate, magnesium sulfate, potassium chloride, and ferrous sulfate), distilled water, and Chloramphenicol	7.3 ± 0.2	25–30 °C for 24–72 h	Selective media for cultivation of superior molds from mixed cultures from food products
Rose Bengal Chloramphenicol agar (RBCA)—Oxoid, UK	Commercial RBCA powder (mycological peptone, glucose, potassium dihydrogen phosphate, agar, magnesium sulfate, Rose Bengal, and Chloramphenicol)	7.2 ± 0.2	25 °C for 24–72 h	Selective media for cultivation of superior molds from mixed cultures from food products (Rose Bengal stops the growth of inferior molds)

**Table 2:** Performance metrics and their corresponding formulas.

Metric	Formula
Sensitivity (True-Positive Rate, TPR)	$\frac{TP}{TP+FN}$
Specificity (True-Negative Rate, TNR)	$\frac{TN}{TN+FP}$
Positive Predictive Value (PPV)	$\frac{TP}{TP+FP}$
Negative Predictive Value (NPV)	$\frac{TN}{TN+FN}$
F1 Score	$\frac{2TP}{2TP+FP+FN}$
Accuracy (ACC)	$\frac{TP+TN}{TP+TN+FP+FN}$

Note: TP = true positives; TN = true negatives; FP = false positives; FN = false negatives.

**Table 3:** Per-species and overall performance metrics on the macroscopic dataset (mean ± standard deviation over 100 trials).

Species	TPR	TNR	PPV	NPV	F1 Score	Accuracy
*Alternaria* spp.	0.9965 ± 0.0201	0.9997 ± 0.0015	0.9983 ± 0.0097	0.9995 ± 0.0030	0.9973 ± 0.0125	–
*Aspergillus Flavi*	0.9988 ± 0.0072	0.9982 ± 0.0071	0.9974 ± 0.0103	0.9992 ± 0.0047	0.9981 ± 0.0072	–
*Aspergillus Nigri*	0.9973 ± 0.0126	0.9996 ± 0.0018	0.9982 ± 0.0086	0.9994 ± 0.0026	0.9977 ± 0.0083	–
*Fusarium* spp.	1.0000 ± 0.0000	1.0000 ± 0.0000	1.0000 ± 0.0000	1.0000 ± 0.0000	1.0000 ± 0.0000	–
Mixed	0.9987 ± 0.0101	0.9996 ± 0.0020	0.9988 ± 0.0061	0.9996 ± 0.0031	0.9987 ± 0.0061	–
*Penicillium* spp.	0.9975 ± 0.0249	0.9999 ± 0.0008	0.9980 ± 0.0199	0.9999 ± 0.0008	0.9975 ± 0.0179	–
*Rhizopus* spp.	0.9800 ± 0.0980	0.9999 ± 0.0008	0.9950 ± 0.0497	0.9997 ± 0.0015	0.9850 ± 0.0749	–
**Overall**	0.9955 ± 0.0186	0.9996 ± 0.0015	0.9979 ± 0.0094	0.9996 ± 0.0013	0.9963 ± 0.0144	0.9979 ± 0.0071

**Table 4:** Per-species and overall performance metrics on the microscopic dataset (mean ± standard deviation over 100 trials).

Species	TPR	TNR	PPV	NPV	F1 Score	Accuracy
*Alternaria* spp.	0.9767 ± 0.1179	0.9997 ± 0.0024	0.9835 ± 0.1093	0.9993 ± 0.0037	0.9781 ± 0.1093	-
*Aspergillus Flavi*	0.9943 ± 0.0165	0.9571 ± 0.1338	0.9774 ± 0.0626	0.9903 ± 0.0277	0.9848 ± 0.0397	-
*Aspergillus Nigri*	0.9790 ± 0.1042	0.9963 ± 0.0121	0.9879 ± 0.0363	0.9948 ± 0.0238	0.9784 ± 0.0959	-
*Fusarium* spp.	0.9175 ± 0.2432	0.9985 ± 0.0066	0.9428 ± 0.2034	0.9930 ± 0.0205	0.9217 ± 0.2302	-
*Penicillium* spp.	0.8960 ± 0.2323	0.9998 ± 0.0015	0.9650 ± 0.1737	0.9945 ± 0.0122	0.9192 ± 0.2039	-
*Rhizopus* spp.	0.9300 ± 0.2551	1.0000 ± 0.0000	0.9300 ± 0.2551	0.9993 ± 0.0027	0.9300 ± 0.2551	-
**Overall**	0.9489 ± 0.1412	0.9919 ± 0.0231	0.9644 ± 0.1209	0.9952 ± 0.0119	0.9520 ± 0.1401	0.9782 ± 0.0577

**Table 5:** Per-species and overall performance metrics on the augmented version of macroscopic dataset.

Species	TPR	TNR	PPV	NPV	F1 Score	Accuracy
*Alternaria* spp.	0.9904 ± 0.0829	0.9896 ± 0.0995	0.9885 ± 0.0872	0.9887 ± 0.1000	0.9832 ± 0.1029	–
*Aspergillus Flavi*	0.9757 ± 0.1336	1.0000 ± 0.0000	0.9900 ± 0.0995	0.9964 ± 0.0190	0.9791 ± 0.1269	–
*Aspergillus Nigri*	0.9863 ± 0.1034	0.9997 ± 0.0020	0.9886 ± 0.0999	0.9975 ± 0.0175	0.9872 ± 0.1008	–
*Fusarium* spp.	0.9890 ± 0.0996	0.9956 ± 0.0380	0.9796 ± 0.1219	0.9983 ± 0.0148	0.9824 ± 0.1118	–
*Mixed*	0.9817 ± 0.1074	0.9997 ± 0.0015	0.9876 ± 0.1003	0.9976 ± 0.0133	0.9843 ± 0.1031	–
*Penicillium* spp.	0.9882 ± 0.1003	0.9972 ± 0.0154	0.9788 ± 0.1123	0.9977 ± 0.0189	0.9829 ± 0.1053	–
*Rhizopus* spp.	0.9765 ± 0.1321	0.9997 ± 0.0016	0.9884 ± 0.0998	0.9971 ± 0.0145	0.9791 ± 0.1242	–
**Overall**	0.9840 ± 0.0975	0.9974 ± 0.0160	0.9859 ± 0.0991	0.9962 ± 0.0273	0.9826 ± 0.1080	0.9841 ± 0.0967

**Table 6:** Per-species and overall performance metrics on the augmented version of microscopic dataset.

Species	TPR	TNR	PPV	NPV	F1 Score	Accuracy
*Alternaria* spp.	0.9878 ± 0.0623	0.9975 ± 0.0082	0.9870 ± 0.0485	0.9975 ± 0.0123	0.9869 ± 0.0536	–
*Aspergillus Flavi*	0.9699 ± 0.0972	0.9974 ± 0.0086	0.9813 ± 0.0809	0.9948 ± 0.0145	0.9748 ± 0.0890	–
*Aspergillus Nigri*	0.9892 ± 0.0472	0.9977 ± 0.0075	0.9889 ± 0.0361	0.9977 ± 0.0115	0.9887 ± 0.0404	–
*Fusarium* spp.	0.9858 ± 0.0363	0.9853 ± 0.0368	0.9451 ± 0.1014	0.9970 ± 0.0080	0.9619 ± 0.0715	–
*Penicillium* spp.	0.9543 ± 0.1178	0.9950 ± 0.0167	0.9716 ± 0.1103	0.9910 ± 0.0232	0.9615 ± 0.1105	–
*Rhizopus* spp.	0.9686 ± 0.0681	0.9981 ± 0.0059	0.9902 ± 0.0308	0.9936 ± 0.0144	0.9782 ± 0.0468	–
**Overall**	0.9759 ± 0.0619	0.9952 ± 0.0123	0.9773 ± 0.0615	0.9953 ± 0.0119	0.9753 ± 0.0653	0.9758 ± 0.0622

**Table 7:** Feature-based comparison with related studies.

Study	Model(s)	Scope	Open Dataset	Code	Evaluation Metrics
[27]	DenseNet, Inception ResNet, InceptionV3, Xception, ResNet50, VGG16, and VGG19	Microscopic identification of yeasts and molds	No	No	ACC, F1, and PPV
[28]	DenseNet-121, InceptionV3, and ResNet-18	*Aspergillus* species identification	No	Yes	ACC, TPR, PPV, and F1
[29]	Random Forest	Tomato-origin macroscopic and microscopic fungal identification	No	No	ACC, TPR, TNR, and F1
[30]	YOLOv8	*Aspergillus* detection on microscopic images	No	No	ACC, PPV, F1, TPR, and IOU
**Our work**	Standard CNN	Macroscopic and microscopic mold identification	**Yes**	**Yes**	TPR, TNR, PPV, NPV, F1, and ACC

## Data Availability

The OpenFungi dataset presented in this article is publicly available for download at https://doi.org/10.5281/zenodo.15692069.

## Abbreviations

The following abbreviations are used in this manuscript:

ACC	Accuracy
AI	Artificial Intelligence
ML	Machine Learning
CNN	Convolutional Neural Network
HIV	Human Immunodeficiency Virus
KOH	Potassium Hydroxide
TPR	True Positive Rate
TNR	True Negative Rate
PPV	Positive Predictive Value
PCR	Polymerase Chain Reaction
MALDI-TOF MS	Matrix-Assisted Laser Desorption/Ionization Time of Flight Mass Spectrometry
NPV	Negative Predictive Value
